# Supplementary material for: Directed differentiation of functional corticospinal-like neurons from endogenous SOX6+/NG2+ cortical progenitors
Source: eLife. 2026 Jan 27;13:RP100340. doi: 10.7554/eLife.100340 (PMC12844902; doi:10.7554/eLife.100340)
Supplement: Supplementary file 3. [file elife-100340-supp3.docx]

**CBIG-NVOF-HA(12,271 bp)**


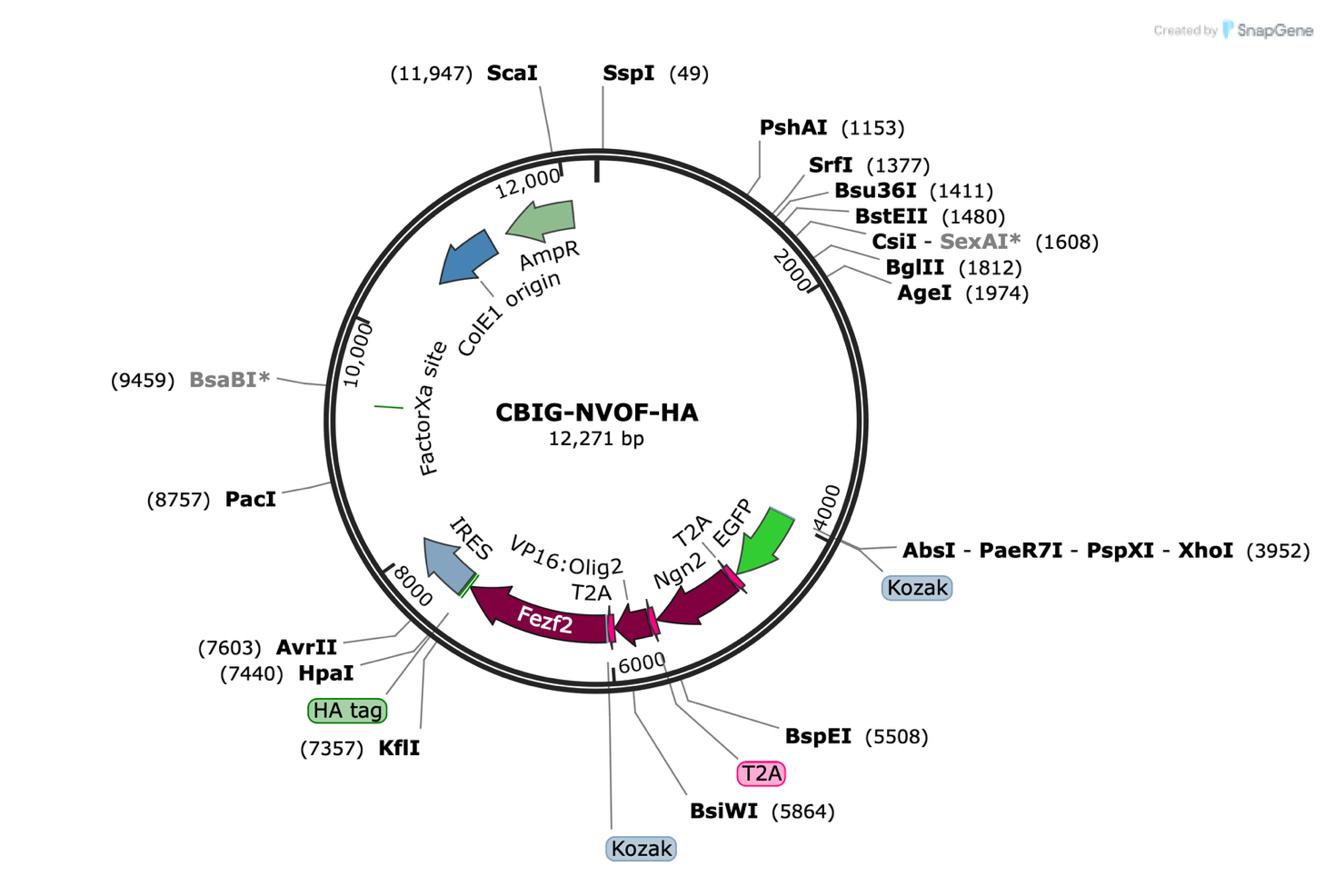


**>CBIG-NVOF-HA(12,271 bp)**

gatccggccattagccatattattcattggttatatagcataaatcaatattggctattggccattgcatacgttgtatc

catatcataatatgtacatttatattggctcatgtccaacattaccgccatgttgacattgattattgactagttattaa

tagtaatcaattacggggtcattagttcatagcccatatatggagttccgcgttacataacttacggtaaatggcccgcc

tggctgaccgcccaacgacccccgcccattgacgtcaataatgacgtatgttcccatagtaacgccaatagggactttcc

attgacgtcaatgggtggagtatttacggtaaactgcccacttggcagtacatcaagtgtatcatatgccaagtacgccc

cctattgacgtcaatgacggtaaatggcccgcctggcattatgcccagtacatgaccttatgggactttcctacttggca

gtacatctacgtattagtcatcgctattaccatggtgatgcggttttggcagtacatcaatgggcgtggatagcggtttg

actcacggggatttccaagtctccaccccattgacgtcaatgggagtttgttttggcaccaaaatcaacgggactttcca

aaatgtcgtaacaactccgccccattgacgcaaatgggcggtaggcatgtacggtgggaggtctatataagcagagctca

ataaaagagcccacaacccctcactcggggcgccagtcctccgattgactgagtcgcccgggtacccgtgtatccaataa

accctcttgcagttgcatccgacttgtggtctcgctgttccttgggagggtctcctctgagtgattgactacccgtcagc

gggggtctttcatttgggggctcgtccgggatcgggagacccctgcccagggaccaccgacccaccaccgggaggtaagc

tggccagcaacttatctgtgtctgtccgattgtctagtgtctatgactgattttatgcgcctgcgtcggtactagttagc

taactagctctgtatctggcggacccgtggtggaactgacgagttcggaacacccggccgcaaccctgggagacgtccca

gggacttcgggggccgtttttgtggcccgacctgagtccaaaaatcccgatcgttttggactctttggtgcacccccctt

agaggagggatatgtggttctggtaggagacgagaacctaaaacagttcccgcctccgtctgaatttttgctttcggttt

gggaccgaagccgcgccgcgcgtcttgtctgctgcagcatcgttctgtgttgtctctgtctgactgtgtttctgtatttg

tctgaaaatatgggcccgggccagactgttaccactcccttaagtttgaccttaggtcactggaaagatgtcgagcggat

cgctcacaaccagtcggtagatgtcaagaagagacgttgggttaccttctgctctgcagaatggccaacctttaacgtcg

gatggccgcgagacggcacctttaaccgagacctcatcacccaggttaagatcaaggtcttttcacctggcccgcatgga

cacccagaccaggtcccctacatcgtgacctgggaagccttggcttttgacccccctccctgggtcaagccctttgtaca

ccctaagcctccgcctcctcttcctccatccgccccgtctctcccccttgaacctcctcgttcgaccccgcctcgatcct

ccctttatccagccctcactccttctctaggcgcccccatatggccatatgagatcttatatggggcacccccgcccctt

gtaaacttccctgaccctgacatgacaagagttactaacagcccctctctccaagctcacttacaggctctctacttagt

ccagcacgaagtctggagacctctggcggcacgtaccaagaacaactggaccgaccggtggtacctcacccttaccgagt

cggcgacacagtgtgggtccgccgacaccagactaagaacctagaacctcgctggaaaggaccttacacagtcctgctga

ccacccccaccgccctcaaagtagacggcatcgcagcttggatacacgccgcccacgtgaaggctgccgaccccgggggt

ggaccatcctctagactgccggatcccagtgtggtggtagggaattctgcaggacttctagttattaatagtaatcaatt

acggggtcattagttcatagcccatatatggagttccgcgttacataacttacggtaaatggcccgcctggctgaccgcc

caacgacccccgcccattgacgtcaataatgacgtatgttcccatagtaacgccaatagggactttccattgacgtcaat

gggtggactatttacggtaaactgcccacttggcagtacatcaagtgtatcatatgccaagtacgccccctattgacgtc

aatgacggtaaatggcccgcctggcattatgcccagtacatgaccttatgggactttcctacttggcagtacatctacgt

attagtcatcgctattaccatgggtcgaggtgagccccacgttctgcttcactctccccatctcccccccctccccaccc

ccaattttgtatttatttattttttaattattttgtgcagcgatgggggcggggggggggggggcgcgcgccaggcgggg

cggggcggggcgaggggcggggcggggcgaggcggagaggtgcggcggcagccaatcagagcggcgcgctccgaaagttt

ccttttatggcgaggcggcggcggcggcggccctataaaaagcgaagcgcgcggcgggcgggagtcgctgcgttgccttc

gccccgtgccccgctccgcgccgcctcgcgccgcccgccccggctctgactgaccgcgttactcccacaggtgagcgggc

gggacggcccttctcctccgggctgtaattagcgcttggtttaatgacggctcgtttcttttctgtggctgcgtgaaagc

cttaaagggctccgggagggccctttgtgcgggggggagcggctcggggggtgcgtgcgtgtgtgtgtgcgtggggagcg

ccgcgtgcggcccgcgctgcccggcggctgtgagcgctgcgggcgcggcgcggggctttgtgcgctccgcgtgtgcgcga

ggggagcgcggccgggggcggtgccccgcggtgcgggggggctgcgaggggaacaaaggctgcgtgcggggtgtgtgcgt

gggggggtgagcagggggtgtgggcgcggcggtcgggctgtaacccccccctgcacccccctccccgagttgctgagcac

ggcccggcttcgggtgcggggctccgtagcggggcgtggcgcggggctcgccgtgccgggcggggggtggcggcaggtgg

gggtgccgggcggggcggggccgcctcgggccggggagggctcgggggaggggcgcggcggccccggagcgccggcggct

gtcgaggcgcggcgagccgcagccattgccttttatggtaatcgtgcgagagggcgcagggacttcctttgtcccaaatc

tggcggagccgaaatctgggaggcgccgccgcaccccctctagcgggcgcgggcgaagcggtgcggcgccggcaggaagg

aaatgggcggggagggccttcgtgcgtcgccgcggcgccgtccccttctccatctccagcctcggggctgccgcaggggg

acggctgccttcgggggggacggggcagggcggggttcggcttctggcgtgtgaccggcggctctagagcctctgctaac

catgttcatgccttcttctttttcctacagctcctgggcaacgtgctggttgttgtgctgtctcatcattttggcaaaga

attgatttcgataccgcgggcccgggatcccCTCGAGGGATCCACCACCATGGTGAGCAAGGGCGAGGAGCTGTTCACCG

GGGTGGTGCCCATCCTGGTCGAGCTGGACGGCGACGTAAACGGCCACAAGTTCAGCGTGTCCGGCGAGGGCGAGGGCGAT

GCCACCTACGGCAAGCTGACCCTGAAGTTCATCTGCACCACCGGCAAGCTGCCCGTGCCCTGGCCCACCCTCGTGACCAC

CCTGACCTACGGCGTGCAGTGCTTCAGCCGCTACCCCGACCACATGAAGCAGCACGACTTCTTCAAGTCCGCCATGCCCG

AAGGCTACGTCCAGGAGCGCACCATCTTCTTCAAGGACGACGGCAACTACAAGACCCGCGCCGAGGTGAAGTTCGAGGGC

GACACCCTGGTGAACCGCATCGAGCTGAAGGGCATCGACTTCAAGGAGGACGGCAACATCCTGGGGCACAAGCTGGAGTA

CAACTACAACAGCCACAACGTCTATATCATGGCCGACAAGCAGAAGAACGGCATCAAGGTGAACTTCAAGATCCGCCACA

ACATCGAGGACGGCAGCGTGCAGCTCGCCGACCACTACCAGCAGAACACCCCCATCGGCGACGGCCCCGTGCTGCTGCCC

GACAACCACTACCTGAGCACCCAGTCCGCCCTGAGCAAAGACCCCAACGAGAAGCGCGATCACATGGTCCTGCTGGAGTT

CGTGACCGCCGCCGGGATCACTCTCGGCATGGACGAGCTGTACAAGGAATTCGTCGAGGAGGGCAGAGGAAGTCTGCTAA

CATGCGGTGACGTCGAGGAGAATCCTGGCCCAGCACCGGGTTCCGTCGAGATGTTCGTCAAATCTGAGACTCTGGAGTTG

AAGGAGGAAGAGGAGGTACTGATGCTGCTGGGCTCGGCTTCCCCGGCCTCGGCGACCCTGACCCCGATGTCCTCCAGCGC

GGACGAGGAGGAGGACGAGGAGCTGCGCCGGCCGGGCTCCGCGCGTGGGCAGCGTGGAGCGGAAGCCGGGCAGGGGGTGC

AGGGCAGTCCGGCGTCGGGTGCCGGGGGTTGCCGGCCAGGGCGGCTGCTGGGCCTGATGCACGAGTGCAAGCGTCGCCCG

TCGCGCTCACGGGCCGTCTCCCGAGGTGCCAAGACGGCGGAGACGGTGCAGCGCATCAAGAAGACCCGCAGGCTCAAGGC

CAACAACCGCGAGCGCAACCGCATGCACAACCTAAACGCCGCGCTGGACGCGCTGCGCGAGGTGCTGCCCACCTTCCCCG

AGGATGCCAAGCTCACGAAGATCGAGACGCTGCGCTTCGCCCACAATTACATCTGGGCGCTCACCGAGACTCTGCGCCTG

GCGGACCACTGCGCCGGCGCCGGTGGCCTCCAGGGGGCGCTCTTCACGGAGGCGGTGCTCCTGAGCCCGGGAGCTGCGCT

CGGCGCCAGCGGGGACAGCCCTTCTCCACCTTCCTCCTGGAGCTGCACCAACAGCCCGGCGTCATCCTCCAACTCCACGT

CCCCATACAGCTGCACTTTATCGCCCGCTAGCCCCGGGTCAGACGTGGACTACTGGCAGCCCCCACCTCCGGAGAAGCAT

CGTTATGCGCCTCACCTGCCCCTCGCCAGGGACTGTATCGTCGAGGAGGGCAGAGGAAGTCTGCTAACATGCGGTGACGT

CGAGGAGAATCCTGGCCCAGCACCGGGTTCCGTCGAGATGTTGGGGGACGGGGATTCCCCGGGGCCGGGATTTACCCCCC

ACGACTCCGCCCCCTACGGCGCTCTGGATATGGCCGACTTCGAGTTTGAGCAGATGTTTACCGATGCCCTTGGAATTGAC

GAGTACGGTGGGGTCGAGCAGCTGCGCCTGAAGATCAACAGCCGCGAACGCAAGCGCATGCACGACCTCAACATCGCCAT

GGACGGCCTGCGGGAGGTCATGCCGTACGCGCACGGGCCGTCGGTGCGCAAGCTCTCCAAGATCGCCACGCTGCTGCTGG

CGCGAAACTACATCCTCATGCTCACCAACTCGCTGGAGGTCGAGGAGGGCAGAGGAAGTCTGCTAACATGCGGTGACGTC

GAGGAGAATCCTGGCCCAGCACCGGGTTCCGTCGAGACCACCATGGCCAGCTCAGCTTCCCTGGAGACCATGGTGCCCCC

GGCCTGCCCGCGCGCTGGAGCGTCACCGGCCACTTCTAAAACACTAGCTTTCTCTATCGAGCGCATCATGGCCAAGACGT

CCGAGCCCCGAGCGCCTTTCGAGCCCCGGCCTGCTGCGTTAGAGGCAGACAGCAGCCAGAGCAAGAAACTGCTCAACCTC

TGCTCGCCGCTGCCCTGTATGATCCCTCTCCAGCCTCTAGGCTACGAGGTGCCGTCCAAGACGCTGCTCAGTTACTCGGA

GTTCTGGAAGAGCAGCCTCCGGGCGGGCGGCGGTGGAGGAGGAGGCAGCGGCGGGGGGGCCCCAGTGTGCGGCGCCAGCG

GCTTGTGCAAAACCAACTGTGGCGTGTGCTGCAAGGCCGAACTGGGCCTCGCGCCTTCTGCGCTGCCCGCCGGCAGGGTC

ATCAAACCGCAGGTCATCAACCAGGCTGTGGGGCTGCCTGCCAGCGGCTCTCTCTACTACTTCAACTACCTGGACTCCAC

CGCTTACCCACCTTCGGAGCTCCTCGGAGGCCACCTTTTCCCATCCGGCCTCCTCAACGCACAGGCCCCCACTTCCCTGG

CTGCTCACCCCAAGCTTTTTCTGCTGGAGAACGCCAAACTGGCCAGCCTGGCTGCGGACAAGTTCCCCCACCCAGCTTCC

TATCCCCATAAGGAGCGCTTGCATGCGCCACTGGAGCAGGTGCTGAAGGAGAACTCGGCCTTGACAGCTGAACGAGGGGG

AGTCAAGAGCCACAGCAAACTACCGGGGGGCTCTACTGACAGCAAACCCAAAAACTTCACCTGCGAAGTGTGCGGCAAGG

TGTTCAATGCTCACTATAACCTCACCCGCCACATGCCTGTCCACACCGGAGCTAGACCGTTTGTGTGCAAAGTCTGTGGC

AAAGGCTTCCGCCAGGCCAGCACTCTCTGCAGACACAAAATTATCCATACCCAGGAAAAACCACATAAGTGTAACCAGTG

CGGCAAAGCCTTCAATCGCAGCTCCACGCTCAACACGCACATCCGCATCCACGCGGGCTACAAGCCCTTCGTCTGCGAGT

TTTGTGGCAAAGGCTTTCACCAAAAAGGGAACTACAAGAATCACAAGCTCACCCACAGCGGCGAGAAGCAGTACAAATGC

ACTATCTGTAACAAGGCCTTCCATCAGGTCTACAATCTGACCTTCCACATGCACACCCACAACGACAAGAAGCCTTTCAC

GTGTGCCACTTGCGGCAAAGGTTTTTGCAGAAACTTTGACTTAAAGAAACATGTGCGCAAACTTCATGACAGCGTGGGTC

CCACCGCCACCCCCTCAGCAAAGGACCTAGCCAGGACAGTTCAGAGCTACCCATACGATGTTCCAGATTACGCTTGAgtt

aacgaattccgcccctctccctcccccccccctaacgttactggccgaagccgcttggaataaggccggtgtgcgtttgt

ctatatgttattttccaccatattgccgtcttttggcaatgtgagggcccggaaacctggccctgtcttcttgacgagca

ttcctaggggtctttcccctctcgccaaaggaatgcaaggtctgttgaatgtcgtgaaggaagcagttcctctggaagct

tcttgaagacaaacaacgtctgtagcgaccctttgcaggcagcggaaccccccacctggcgacaggtgcctctgcggcca

aaagccacgtgtataagatacacctgcaaaggcggcacaaccccagtgccacgttgtgagttggatagttgtggaaagag

tcaaatggctctcctcaagcgtattcaacaaggggctgaaggatgcccagaaggtaccccattgtatgggatctgatctg

gggcctcggtgcacatgctttacatgtgtttagtcgaggttaaaaaacgtctaggccccccgaaccacggggacgtggtt

ttcctttgaaaaacacgatgataatatggccacaaccaagggcgaggagctgttcaccggggtggtgcccatcctggtcg

agctggacggcgacgtgaacggccacaagttcagcgtgtccggcgagggcgagggcgatgccacctacggcaagctgacc

ctgaagttcatctgcaccaccggcaagctgcccgtgccctggcccaccctcgtgaccaccctgacctacggcgtgcagtg

cttcagccgctaccccgaccacatgaagcagcacgacttcttcaagtccgccatgcccgaaggctacgtccaggagcgca

ccatcttcttcaaggacgacggcaactacaagacccgcgccgaggtgaagttcgagggcgacaccctggtgaaccgcatc

gagctgaagggcatcgacttcaaggaggacggcaacatcctggggcacaagctggagtacaactacaacagccacaacgt

ctatatcatggccgacaagcagaagaacggcatcaagcgcaacttcaagatccgccacaacatcgaggacggcagcgtgc

agctcgccgaccactaccagcagaacacccccatcggcgacggccccgtgctgctgcccgacaaccactacctgagcacc

cagtccgccctgagcaaagaccccaacgagaagcgcgatcacatggtcctgctggagttcgtgaccgccgccgggatcac

tcacggcatggacgagctgtacaagtaatgaattaattaagaattatcaccgcttctattcagccagtaaggcctgtctt

taatggcctccggcatgagacactttctagagtctgaggaattcgatatcaagcttgatcgataatcaacctctggatta

caaaatttgtgaaagattgactggtattcttaactatgttgctccttttacgctatgtggatacgctgctttaatgcctt

tgtatcatgctattgcttcccgtatggctttcattttctcctccttgtataaatcctggttgctgtctctttatgaggag

ttgtggcccgttgtcaggcaacgtggcgtggtgtgcactgtgtttgctgacgcaacccccactggttggggcattgccac

cacctgtcagctcctttccgggactttcgctttccccctccctattgccacggcggaactcatcgccgcctgccttgccc

gctgctggacaggggctcggctgttgggcactgacaattccgtggtgttgtcggggaagctgacgtcctttccatggctg

ctcgcctgtgttgccacctggattctgcgcgggacgtccttctgctacgtcccttcggccctcaatccagcggaccttcc

ttcccgcggcctgctgccggctctgcggcctcttccgcgtcttcgccttcgccctcagacgagtcggatctccctttggg

ccgcctccccgcctgatcgatatcgatgtcgacgaattccagctgagcgccggtcgctaccattaccagttggtctggtg

tcaaaaataataataaccgggcaggccatgtctgcccgtatttcgcgtaaggaaatccattatgtactattttcgagcgg

ccgccagcacagtggtcgacgataaaataaaagattttatttagtctccagaaaaaggggggaatgaaagaccccacctg

taggtttggcaagctagcttaagtaacgccattttgcaaggcatggaaaaatacataactgagaatagagaagttcagat

caaggtcaggaacagatggaacagctgaatatgggccaaacaggatatctgtggtaagcagttcctgccccggctcaggg

ccaagaacagatggaacagctgaatatgggccaaacaggatatctgtggtaagcagttcctgccccggctcagggccaag

aacagatggtccccagatgcggtccagccctcagcagtttctagagaaccatcagatgtttccagggtgccccaaggacc

tgaaatgaccctgtgccttatttgaactaaccaatcagttcgcttctcgcttctgttcgcgcgcttctgctccccgagct

caataaaagagcccacaacccctcactcggggcgccagtcctccgattgactgagtcgcccgggtacccgtgtatccaat

aaaccctcttgcagttgcatccgacttgtggtctcgctgttccttgggagggtctcctctgagtgattgactacccgtca

gcgggggtctttcatttccgacttgtggtctcgctgccttgggagggtctcctctgagtgattgactacccgtcagcggg

ggtcttcacatgcagcatgtatcaaaattaatttggttttttttcttaagtatttacattaaatggccatagttgcatta

atgaatcggccaacgcgcggggagaggcggtttgcgtattggcgctcttccgcttcctcgctcactgactcgctgcgctc

ggtcgttcggctgcggcgagcggtatcagctcactcaaaggcggtaatacggttatccacagaatcaggggataacgcag

gaaagaacatgtgagcaaaaggccagcaaaaggccaggaaccgtaaaaaggccgcgttgctggcgtttttccataggctc

cgcccccctgacgagcatcacaaaaatcgacgctcaagtcagaggtggcgaaacccgacaggactataaagataccaggc

gtttccccctggaagctccctcgtgcgctctcctgttccgaccctgccgcttaccggatacctgtccgcctttctccctt

cgggaagcgtggcgctttctcatagctcacgctgtaggtatctcagttcggtgtaggtcgttcgctccaagctgggctgt

gtgcacgaaccccccgttcagcccgaccgctgcgccttatccggtaactatcgtcttgagtccaacccggtaagacacga

cttatcgccactggcagcagccactggtaacaggattagcagagcgaggtatgtaggcggtgctacagagttcttgaagt

ggtggcctaactacggctacactagaaggacagtatttggtatctgcgctctgctgaagccagttaccttcggaaaaaga

gttggtagctcttgatccggcaaacaaaccaccgctggtagcggtggtttttttgtttgcaagcagcagattacgcgcag

aaaaaaaggatctcaagaagatcctttgatcttttctacggggtctgacgctcagtggaacgaaaactcacgttaaggga

ttttggtcatgagattatcaaaaaggatcttcacctagatccttttaaattaaaaatgaagtttgcggccgcaaatcaat

ctaaagtatatatgagtaaacttggtctgacagttaccaatgcttaatcagtgaggcacctatctcagcgatctgtctat

ttcgttcatccatagttgcctgactccccgtcgtgtagataactacgatacgggagggcttaccatctggccccagtgct

gcaatgataccgcgagacccacgctcaccggctccagatttatcagcaataaaccagccagccggaagggccgagcgcag

aagtggtcctgcaactttatccgcctccatccagtctattaattgttgccgggaagctagagtaagtagttcgccagtta

atagtttgcgcaacgttgttgccattgctacaggcatcgtggtgtcacgctcgtcgtttggtatggcttcattcagctcc

ggttcccaacgatcaaggcgagttacatgatcccccatgttgtgcaaaaaagcggttagctccttcggtcctccgatcgt

tgtcagaagtaagttggccgcagtgttatcactcatggttatggcagcactgcataattctcttactgtcatgccatccg

taagatgcttttctgtgactggtgagtactcaaccaagtcattctgagaatagtgtatgcggcgaccgagttgctcttgc

ccggcgtcaacacgggataataccgcgccacatagcagaactttaaaagtgctcatcattggaaaacgttcttcggggcg

aaaactctcaaggatcttaccgctgttgagatccagttcgatgtaacccactcgtgcacccaactgatcttcagcatctt

ttactttcaccagcgtttctgggtgagcaaaaacaggaaggcaaaatgccgcaaaaaagggaataagggcgacacggaaa

tgttgaatactcatactcttcctttttcaat
